# Editorial: Diabetes management through lifestyle and the social determinants of health

**DOI:** 10.3389/fnut.2023.1158322

**Published:** 2023-05-10

**Authors:** Lubia Velázquez López, Abril Violeta Muñoz Torres, Miguel Klünder Klünder, Oswaldo Sinoe Medina Gómez

**Affiliations:** ^1^Unidad de Investigación en Epidemiología Clínica, Hospital General Regional No 1, “Dr. Carlos Mac Gregor Sánchez Navarro” Instituto Mexicano del Seguro Social, Ciudad de México, Mexico; ^2^Departamento de Salud Publica, Escuela de Medicina, Universidad Nacional Autónoma de México, Ciudad de México, Mexico; ^3^Departamento de Investigación, Hospital Infantil de México Federico Gómez, Ciudad de México, Mexico

**Keywords:** diabetes type 2, lifestyle, social determinants of health, diabetes management, diabetes prevention

A healthy lifestyle has been associated with the prevention of diabetes and its complications, as well as with achieving metabolic goals and a lower risk of all-cause mortality in patients with type 2 diabetes (1, 2).

Lifestyle includes aspects such as diabetes self-management education, nutritional therapy, and physical activity. These aspects are considered an integral part of the treatment of patients with diabetes (3).

The objective of this Research Topic is to present articles describing the effect of lifestyle-associated variables, such as medical nutrition therapy, on diabetes management. This topic highlights the importance of some diet components, such as fiber and magnesium content, food groups, and macronutrient distribution for diabetes prevention or disease care.

This topic also addresses the social determinants of health and their association with diabetes prevention and access to health care in vulnerable populations.

In patients with type 2 diabetes, a higher dietary fiber intake has been identified as favorable for improving glycemic control and lipid levels, reducing body weight, and reducing premature all-cause mortality (4). In this sense, Takahashi et al. showed in a cross-sectional study of 260 men and 200 women the positive correlation between higher fiber intake and skeletal muscle mass and muscle-to-fat ratio, as well as a negative correlation with body fat mass in women with type 2 diabetes. Longitudinal studies have contributed to studying the benefit of dietary fiber intake on the body composition of patients with type 2 diabetes.

One of the main limitations of medical nutrition therapy is adherence to a healthy diet so to improve adherence to the diet, it should be adapted to each region, its tastes, customs, and economy (5). The study reported by Hamdy et al. shows the cross-cultural adjustment of their Diabetes Nutrition Algorithm (tDNA) group. The results provide a compendium of the guidelines established to provide nutritional counseling adapted to the culture of Middle Eastern countries and contribute to providing nutritional guidance adapted to the region to improve diet adherence. The association of dietary patterns with the incidence of diabetes has been previously evaluated, where it has been identified that dietary patterns, such as the Dietary Approaches to Stop Hypertension (DASH) diet and the Alternative Healthy Eating Index, reduce the incidence of diabetes risk (6). In addition, there is evidence of a lower risk of developing the disease with higher consumption of fruits and vegetables and less intake of processed meat, sugar-sweetened beverages, and refined grains (7). The dietary plan for patients with diabetes should be personalized; the benefit of incorporating snacks rich in protein and monounsaturated fats, such as nuts and almonds, has been previously reported and helps achieve better control of glucose variability throughout the day. This nutritional strategy is still under research. Liu et al. proposed a protocol for a clinical trial with 32 volunteers with prediabetes who, distributed into 4 groups, will receive interventions with different types of foods to evaluate their effect on glycemic variability.

One of the goals of lifestyle strategies is to delay or reduce the development of diabetes. Hosseini-Esfahani et al. performed a secondary analysis on 6,112 subjects who were selected from the Tehran lipid and glucose study (8). Their diet was assessed through a semiquantitative food questionnaire, and the incidence of type 2 diabetes was calculated. The results showed that a diet with higher consumption of legumes significantly reduces the risk of type 2 diabetes when compared to a diet rich in poultry (Hosseini-Esfahani et al.).

In a population study, Huang et al. reported a risk twice as high of presenting type 2 diabetes in a population exposed to pesticides at home. There was an association between a higher magnesium content in the diet and a lower risk of developing diabetes in the population exposed to pesticides.

The association between a higher magnesium content in the diet and a lower risk for the development of diabetes has been previously evidenced; however, the relationship between pesticide exposure, dietary magnesium intake, and diabetes development needs to be supported by longitudinal studies (9).

Social determinants of health refer to the social circumstances or conditions in which people are born, grow, live, work, and age, as well as the health care system. These conditions generate inequalities in health care. Evidence suggests a relationship between low SDH and risk of diabetes, poor glycemic control, low health literacy, depressive symptoms, and difficulty obtaining care (10).

In this sense, Hernández-Montoya et al. studied 371 adolescents from hard-to-reach localities in Mexico who had a lack of access to health services and perceived vulnerability. The authors found that 61.9% of the subjects were diagnosed in the study with prediabetes and had a higher HbA1c level compared to a subsample from a different higher social stratum group (Hernández-Montoya et al.). Life conditions such as poverty or social inequality and ethnicity or race are factors that could be associated with the development of diabetes, particularly when conditioned by the lifestyle of the population (11).

The objectives of interventions aimed at adopting a healthy lifestyle in patients with diabetes are comprehensive metabolic control and the influence of protective levels of HbA1c, lipids, blood pressure, and body weight to prevent complications of the disease. Within the subtopic diet, Pan et al. identified a positive association between a proinflammatory-type diet and the prevalence of retinopathy in 2,403 participants with diabetes.

All submitted manuscripts contribute to the evidence of lifestyle variables in prevention and diabetes management; however, further studies that consider other exposure variables, such as determinants of health, are still needed to evaluate different types of dietary patterns, dietary interventions, educational strategies, and interventions with the use of technology.

## Author contributions

LV wrote the first draft of the manuscript. AM, MK, and OG wrote sections of the manuscript. All authors contributed to the article and approved the submitted version.
